# Hippo Signaling Dysregulation in Breast Cancer: Subtype-Independent Gene and miRNA Signatures

**DOI:** 10.3390/biomedicines13102342

**Published:** 2025-09-25

**Authors:** Katarzyna Król-Jatręga, Elżbieta Mitka-Krysiak, Kacper Boroń, Nikola Zmarzły, Piotr Ossowski, Aleksandra Plata-Babula, Paweł Ordon, Wojciech Kulej, Tomasz Sirek, Julia Gajdeczka, Yuriy Prudnikov, Krzysztof Bereza, Olga Nowotny-Czupryna, Dariusz Boroń, Beniamin Oskar Grabarek

**Affiliations:** 1 Collegium Medicum, WSB University, 41-300 Dabrowa Gornicza, Poland; elzbietamitkakrysiak@gmail.com (E.M.-K.); nikola.zmarzly@gmail.com (N.Z.); drpiotrossowski@gmail.com (P.O.); draplatababula@gmail.com (A.P.-B.); wojciechkulej@hotmail.com (W.K.); drtstierka@gmail.com (T.S.); gajdeczkajulia@gmail.com (J.G.); yuriy.prudnikov@wsb.edu.pl (Y.P.); onowotny-czupryna@wsb.edu.pl (O.N.-C.); dariusz@boron.pl (D.B.); bgrabarek7@gmail.com (B.O.G.); 2 Department of Plastic Surgery, Faculty of Medicine, Academia of Silesia, 40-555 Katowice, Poland; q375@icloud.com; 3 Department of Plastic and Reconstructive Surgery, Hospital for Minimally Invasive and Reconstructive Surgery in Bielsko-Biała, 43-316 Bielsko-Biala, Poland; 4 Department of Mother and Child Health, Faculty of Health Sciences, Institute of Nursing and Midwifery, Jagiellonian University Medical College, 31-008 Cracow, Poland; 5 Faculty of Medicine and Health Sciences, Andrzej Frycz Modrzewski University in Kraków, 30-705 Cracow, Poland; 6 Department of Gynecology and Obstetrics, TOMMED Specjalisci od Zdrowia, 40-851 Katowice, Poland; 7 Department of Gynecology and Obstetrics with Gynecologic Oncology, Ludwik Rydygier Memorial Specialized Hospital, 31-826 Cracow, Poland

**Keywords:** breast cancer, miRNA, Hippo pathway

## Abstract

**Background/Objectives**: Breast cancer represents a diverse group of malignancies and continues to rank among the leading causes of cancer-related deaths in women. Altered Hippo pathway signaling has been increasingly recognized as a contributor to tumor growth, therapeutic resistance, and metastatic spread. This study aimed to identify miRNAs targeting Hippo pathway-related genes that are consistently dysregulated across all five breast cancer subtypes. **Methods**: The study cohort included patients representing five breast cancer subtypes: 130 luminal A, 96 HER2-positive luminal B, 100 HER2-negative luminal B, 36 non-luminal HER2-positive, and 43 triple-negative breast cancer (TNBC). Tumor samples were collected during surgery, along with adjacent healthy tissue that served as controls. Expression of Hippo-related genes was analyzed using mRNA microarrays and validated with reverse transcription quantitative polymerase chain reaction (RT-qPCR). Protein levels were assessed via enzyme-linked immunosorbent assay (ELISA), while miRNA expression profiling was performed with miRNA microarrays. Potential mRNA targets were predicted using the miRDB database. **Results**: We identified consistent downregulation of *STK4*, *RASSF6*, and *FGF1*, alongside overexpression of *BIRC5* and *SERPINE1*. miRNA analysis revealed that *STK4* is potentially regulated by miR-522-3p, *SERPINE1* by miR-199b-5p and miR-30a-3p, whereas *RASSF6*, *FGF1*, and *BIRC5* appeared to be predominantly regulated at the transcriptional level. These alterations reflect both the suppression of upstream Hippo activation and activation of downstream oncogenic effectors across all subtypes. **Conclusions**: Our findings reveal a conserved Hippo dysregulation program in breast cancer, highlighting subtype-independent Hippo-related genes and their miRNA regulators as potential universal biomarkers and therapeutic targets, complementing subtype-specific treatment strategies.

## 1. Introduction

Breast cancer continues to be the most common malignancy diagnosed in women and the foremost cause of cancer mortality worldwide [1]. It significantly impacts patients beyond survival by causing long-term morbidity, reducing quality of life through physical, psychological, and financial burdens and imposing substantial economic costs on healthcare systems and societies [2]. Breast cancer represents a biologically diverse disease composed of different molecular subtypes: luminal A, luminal B HER2-positive, luminal B HER2-negative, non-luminal HER2-positive, and triple-negative breast cancer (TNBC). Each subtype is defined by specific transcriptomic features, clinical behavior, and therapeutic approaches [3].

The Hippo signaling pathway is a fundamental regulator of tissue homeostasis, controlling cell proliferation, differentiation, and apoptosis [4]. Its core components include the serine/threonine-protein kinases 4/3 (STK4/3) and the adaptor Salvador (SAV1), which activate the large tumor suppressor kinases 1/2 (LATS1/2) through phosphorylation. In cooperation with MOB kinase activators 1A and 1B (MOB1A/B), activated LATS1/2 phosphorylates the transcriptional co-activators Yes-associated protein (YAP) and transcriptional coactivator with PDZ-binding motif (TAZ), leading to their cytoplasmic retention and degradation [5]. When Hippo signaling is functional, nuclear translocation of YAP/TAZ and activation of growth-promoting genes is suppressed [6]. In contrast, Hippo inactivation enables nuclear accumulation of YAP and TAZ, where they interact with transcriptional enhanced associate domain (TEAD) transcription factors to activate gene programs promoting cell proliferation, resistance to apoptosis, migration, and epithelial–mesenchymal transition (EMT) [7,8].

Altered Hippo signaling has therefore emerged as a hallmark of oncogenesis, contributing not only to tumor initiation but also to therapy resistance and metastatic progression [9]. The pathway is not limited to its canonical kinase cascade but also integrates multiple upstream signals, including extracellular matrix stiffness, G protein-coupled receptor signaling, and growth factor pathways [10,11]. Through this integration, Hippo interacts with several major oncogenic pathways, such as Wnt/β-catenin, TGF-β, PI3K/AKT, and MAPK [12,13,14,15]. This positioning highlights Hippo as both a driver of tumor cell-intrinsic behavior and a mediator of tumor-microenvironment interactions.

MicroRNAs (miRNAs) provide an additional layer of regulation by modulating gene expression at the post-transcriptional level. Dysregulated miRNA activity has been linked to breast cancer development, prognosis, and treatment resistance [16]. Yet, their contribution to Hippo signaling across breast cancer subtypes is not fully understood.

This study aimed to identify miRNAs that target Hippo pathway-related genes consistently dysregulated across all five breast cancer subtypes.

## 2. Materials and Methods

### 2.1. Patients

A total of 405 patients were included in the cohort. Obtained samples were classified as luminal A (130 cases), HER2-negative luminal B (100 cases), HER2-positive luminal B (96 cases), non-luminal HER2-positive (36 cases), and triple-negative breast cancer (TNBC; 43 cases). Control samples from adjacent non-cancerous tissue were also obtained intraoperatively. Every patient was staged as T1N0M0. Across all subtypes, the majority of patients were older than 50 years, with an average BMI above 30 (Table 1) [17].

The study protocol complied with the ethical guidelines of the Declaration of Helsinki and was approved by the Bioethical Committee of the Regional Medical Chamber in Krakow (81/KBL/OIL/2023; 10 March 2023). All participants provided written informed consent.

### 2.2. Isolation of Total RNA

Isolation of total RNA was performed using TRIzol Reagent (Invitrogen Life Technologies, Carlsbad, CA, USA; cat. no. 15596026). Subsequent purification was carried out with the RNeasy Mini Kit (QIAGEN, Hilden, Germany; cat. no. 74104), followed by DNase I treatment (Fermentas International Inc., Burlington, ON, Canada; cat. no. 18047019). RNA integrity and yield were verified by 1% agarose gel electrophoresis and spectrophotometry.

### 2.3. mRNA Profiling by Microarrays

HG-U133A 2.0 microarrays (Affymetrix, Santa Clara, CA, USA) in combination with the GeneChip™ 3′ IVT PLUS kit (Thermo Fisher Scientific, Waltham, MA, USA; cat. no. 902416) were employed for transcriptome profiling. Genes linked to the Hippo signaling pathway were retrieved from the Kyoto Encyclopedia of Genes and Genomes (KEGG) pathway database (hsa04390), yielding 157 genes mapped to 411 mRNA probes on the platform.

### 2.4. Gene Expression Analysis by Reverse Transcription Quantitative Polymerase Chain Reaction (RT-qPCR)

Validation of microarray data was carried out by RT-qPCR employing the SensiFast SYBR No-ROX One-Step Kit (Bioline, London, UK). Five candidate genes (*BIRC5*, *FGF1*, *RASSF6*, *SERPINE1*, *STK4*; Table 2) were analyzed. Relative expression was determined using the 2^−ΔΔCt^ method with β-actin (ACTB) as the reference gene.

### 2.5. Enzyme-Linked Immunosorbent Assay (ELISA)

Protein expression was assessed via ELISA with MyBioSource (San Diego, CA, USA) kits targeting BIRC5 (cat. no. MBS2887028), FGF1 (cat. no. MBS167170), RASSF6 (cat. no. MBS7612748), SERPINE1 (cat. no. MBS763621), STK4 (cat. no. MBS167492).

### 2.6. miRNA Profiling and Target Prediction

miRNA Microarray 2.0, in combination with the FlashTag Biotin HSR RNA Labeling Kit and the Hybridization Wash and Stain Kit (Affymetrix, Santa Clara, CA, USA), was utilized to detect miRNAs differentiating breast cancer from control tissue. The miRDB database (http://mirdb.org; accessed on 2 August 2025) was employed to predict regulatory links between miRNAs and the selected genes (*BIRC5*, *FGF1*, *RASSF6*, *SERPINE1, STK4*), considering only targets with a prediction score ≥ 80 [18].

### 2.7. Statistical Analysis

Transcriptome Analysis Console (Thermo Fisher Scientific, Waltham, MA, USA) was used for microarray data processing. One-way ANOVA and Tukey’s post hoc test were applied with thresholds set at *p* < 0.05 and FC > 2 or <−2. Gene lists for each breast cancer subtype and their intersections were generated using Python (ver. 3.13.7). Venn diagram was created in R (ver. 4.4.0; package Venn ver. 1.12). Final figures were assembled in Adobe Photoshop. For RT-qPCR and ELISA data, Statistica 13.3 (StatSoft, Krakow, Poland) was employed. Data distribution was verified with the Shapiro-Wilk test. The Kruskal-Wallis test and Dunn’s post hoc test were then carried out.

Overall survival (OS) across breast cancer subtypes was assessed using the Kaplan-Meier plotter (http://kmplot.com/, accessed on 28 June 2025), applying a 60-month follow-up cut-off [19,20].

Sample size estimation with G*Power 3.1.9.718 (f = 0.25, α = 0.05, power = 0.95) yielded *n* = 324, while the study’s 405 participants provided a post hoc power of 0.98 [21].

## 3. Results

### 3.1. mRNA Microarray-Based Gene Expression Profiling

**Figure 1:**
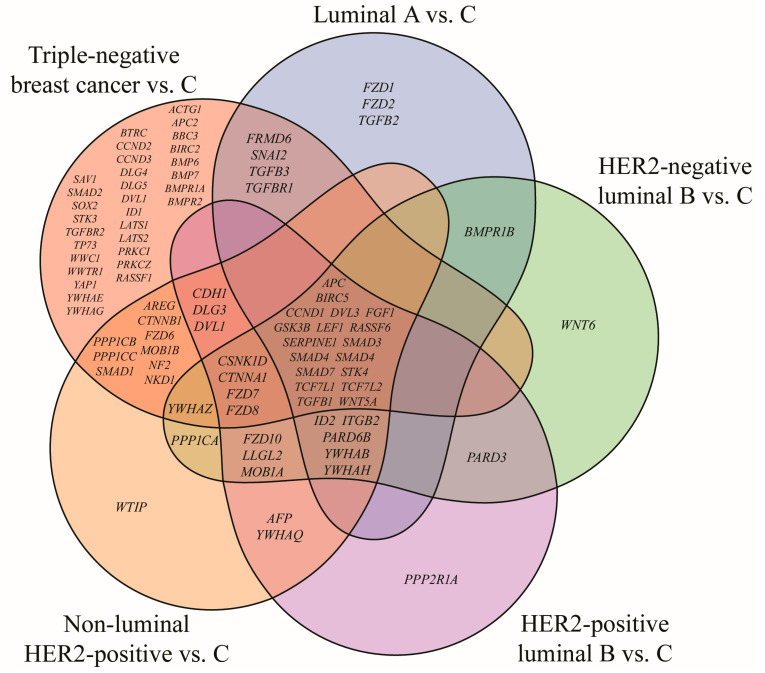
Venn diagram of Hippo pathway genes significantly altered in breast cancer subtypes compared with the control (*p* < 0.05; FC > 2 or < −2). HER2, human epidermal growth factor receptor 2; C, control; *ACTG1*, actin gamma 1; *AFP*, alpha fetoprotein; *APC*, APC regulator of WNT signaling pathway; *APC2*, APC regulator of WNT signaling pathway 2; *AREG*, amphiregulin; *BBC3*, BCL2 binding component 3; *BIRC2*, baculoviral IAP repeat containing 2; *BIRC5*, baculoviral IAP repeat containing 5; *BMP6*, bone morphogenetic protein 6; *BMP7*, bone morphogenetic protein 7; *BMPR1A*, bone morphogenetic protein receptor type 1A; *BMPR1B*, bone morphogenetic protein receptor type 1B; *BMPR2*, bone morphogenetic protein receptor type 2; *BTRC*, beta-transducin repeat containing E3 ubiquitin protein ligase; *CCND1*, cyclin D1; *CCND2*, cyclin D2; *CCND3*, cyclin D3; *CDH1*, cadherin 1; *CSNK1D*, casein kinase 1 delta; *CTNNA1*, catenin alpha 1; *CTNNB1*, catenin beta 1; *DLG1*, discs large MAGUK scaffold protein 1; *DLG3*, discs large MAGUK scaffold protein 3; *DLG4*, discs large MAGUK scaffold protein 4; *DLG5*, discs large MAGUK scaffold protein 5; *DVL1*, dishevelled segment polarity protein 1; *DVL3*, dishevelled segment polarity protein 3; *FGF1*, fibroblast growth factor 1; *FRMD6*, FERM domain containing 6; *FZD1*, frizzled class receptor 1; *FZD2*, frizzled class receptor 2; *FZD3*, frizzled class receptor 3; *FZD4*, frizzled class receptor 4; *FZD6*, frizzled class receptor 6; *FZD7*, frizzled class receptor 7; *FZD8*, frizzled class receptor 8; *FZD10*, frizzled class receptor 10; *GSK3B*, glycogen synthase kinase 3 beta; *ID1*, inhibitor of DNA binding 1; *ID2*, inhibitor of DNA binding 2; *ITGB2*, integrin subunit beta 2; *LATS1*, large tumor suppressor kinase 1; *LATS2*, large tumor suppressor kinase 2; *LEF1*, lymphoid enhancer binding factor 1; *LLGL2*, LLGL scribble cell polarity complex component 2; *MOB1A*, MOB kinase activator 1A; *MOB1B*, MOB kinase activator 1B; *NF2*, moesin-ezrin-radixin like (MERLIN) tumor suppressor; *NKD1*, NKD inhibitor of WNT signaling pathway 1; *PARD3*, par-3 family cell polarity regulator; *PARD6B*, par-6 family cell polarity regulator beta; *PPP1CA*, protein phosphatase 1 catalytic subunit alpha; *PPP1CB*, protein phosphatase 1 catalytic subunit beta; *PPP1CC*, protein phosphatase 1 catalytic subunit gamma; *PPP2R1A*, protein phosphatase 2 scaffold subunit Aalpha; *PRKCI*, protein kinase C iota; *PRKCZ*, protein kinase C zeta; *RASSF1*, Ras association domain family member 1; *RASSF6*, Ras association domain family member 6; *SAV1*, salvador family WW domain containing protein 1; *SERPINE1*, serpin family E member 1; *SMAD1*, SMAD family member 1; *SMAD2*, SMAD family member 2; *SMAD3*, SMAD family member 3; *SMAD4*, SMAD family member 4; *SMAD7*, SMAD family member 7; *SNAI2*, snail family transcriptional repressor 2; *SOX2*, SRY-box transcription factor 2; *STK3*, serine/threonine kinase 3; *STK4*, serine/threonine kinase 4; *TCF7L1*, transcription factor 7 like 1; *TCF7L2*, transcription factor 7 like 2; *TGFB1*, transforming growth factor beta 1; *TGFB2*, transforming growth factor beta 2; *TGFB3*, transforming growth factor beta 3; *TGFBR1*, transforming growth factor beta receptor 1; *TGFBR2*, transforming growth factor beta receptor 2; *TP73*, tumor protein p73; *WNT5A*, Wnt family member 5A; *WNT6*, Wnt family member 6; *WTIP*, WT1 interacting protein; *WWC1*, WW and C2 domain containing 1; *WWTR1*, WW domain containing transcription regulator 1; *YAP1*, Yes1 associated transcriptional regulator; *YWHAB*, tyrosine 3-monooxygenase/tryptophan 5-monooxygenase activation protein beta; *YWHAE*, tyrosine 3-monooxygenase/tryptophan 5-monooxygenase activation protein epsilon; *YWHAG*, tyrosine 3-monooxygenase/tryptophan 5-monooxygenase activation protein gamma; *YWHAH*, tyrosine 3-monooxygenase/tryptophan 5-monooxygenase activation protein eta; *YWHAQ*, tyrosine 3-monooxygenase/tryptophan 5-monooxygenase activation protein theta; *YWHAZ*, tyrosine 3-monooxygenase/tryptophan 5-monooxygenase activation protein zeta.

Among the 418 mRNAs associated with the Hippo signaling pathway, 145 were found to be significantly dysregulated in breast cancer compared with control tissue. Subsequent Tukey’s post hoc testing demonstrated subtype-specific expression changes, with 45 transcripts altered in luminal A, 52 in HER2-negative luminal B, 58 in HER2-positive luminal B, 70 in non-luminal HER2-positive, and 117 in TNBC. The overlapping and unique genes among the subtypes are depicted in the Venn diagram (Figure 1).

The analysis identified subtype-specific genes, with TNBC displaying the largest number. In addition, 18 genes were common to all breast cancer cases irrespective of subtype. 13 of these have been reported in our previous studies, while the current study concentrated on five novel genes: *BIRC5*, *FGF1*, *RASSF6*, *SERPINE1* and *STK4* (Table 3).

Expression profiling indicated upregulation of *BIRC5* and *SERPINE1* independent of breast cancer subtype. In contrast, *FGF1*, *RASSF6* and *STK4* exhibited significant downregulation in this cohort.

### 3.2. RT-qPCR and ELISA Analysis of BIRC5, FGF1, RASSF6, SERPINE1 and STK4 Expression

**Figure 2:**
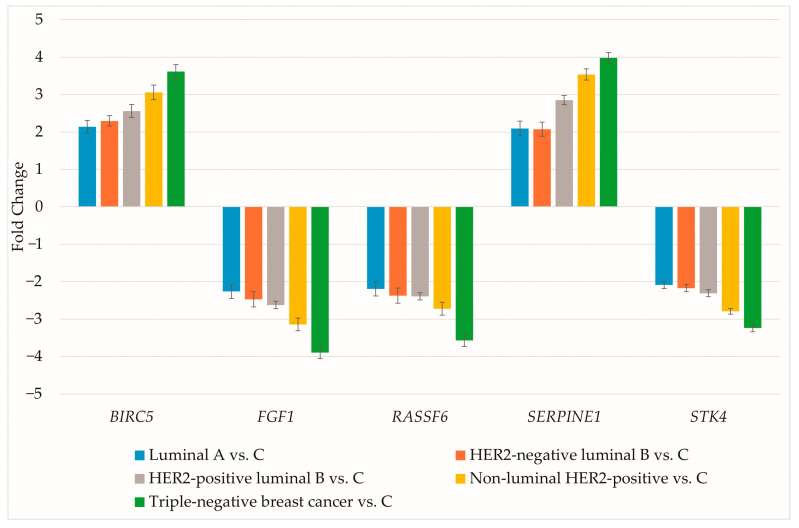
RT-qPCR assessment of genes differentiating breast cancer regardless of subtype. HER2, human epidermal growth factor receptor 2; C, control; *BIRC5*, baculoviral IAP repeat containing 5; *FGF1*, fibroblast growth factor 1; *RASSF6*, Ras association domain family member 6; *SERPINE1*, serpin family E member 1; *STK4*, serine/threonine kinase 4.

The expression of genes differentiating breast cancer irrespective of subtype was evaluated using RT-qPCR. Results are presented as mean ± standard deviation (Figure 2).

RT-qPCR data aligned with the microarray findings. Protein concentrations for the selected genes were then determined (Table 4).

The results obtained from protein quantification were consistent with those observed for mRNA expression.

### 3.3. Prediction of miRNA Targets

The subsequent step involved evaluating whether *BIRC5*, *FGF1*, *RASSF6*, *SERPINE1* and *STK4* could serve as targets for miRNAs differentiating breast cancer from the control (Table 5).

*BIRC5*, *FGF1* and *RASSF6* are unlikely to be targets of miRNAs significantly altered across five breast cancer subtypes in this study. *SERPINE1* overexpression may be associated with low levels of miR-199b-5p and miR-30a-3p. Moreover, decreased expression of *STK4* may be the result of high miR-522-3p activity.

### 3.4. Overall Survival Outcomes Across Breast Cancer Subtypes

**Figure 3:**
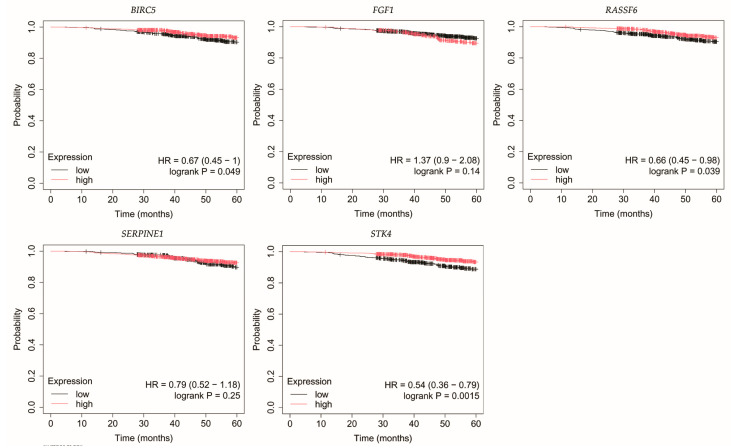
Overall survival of patients with luminal A breast cancer. *BIRC5*, baculoviral IAP repeat containing 5; *FGF1*, fibroblast growth factor 1; *RASSF6*, Ras association domain family member 6; *SERPINE1*, serpin family E member; *STK4*, serine/threonine kinase 4.

**Figure 4:**
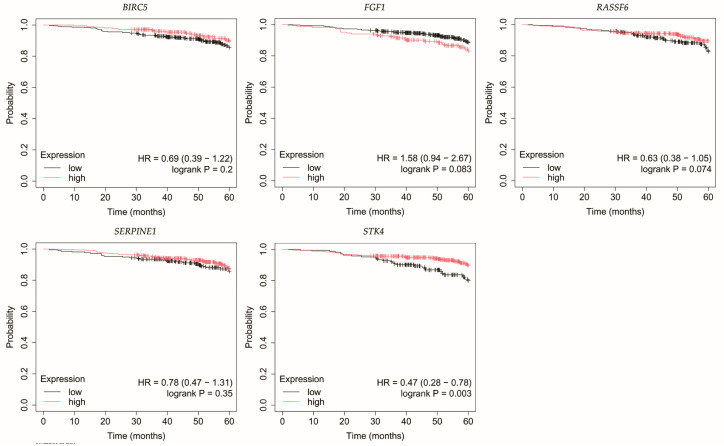
Overall survival of patients with HER2-negative luminal B breast cancer. *BIRC5*, baculoviral IAP repeat containing 5; *FGF1*, fibroblast growth factor 1; *RASSF6*, Ras association domain family member 6; *SERPINE1*, serpin family E member 1; *STK4*, serine/threonine kinase 4.

**Figure 5:**
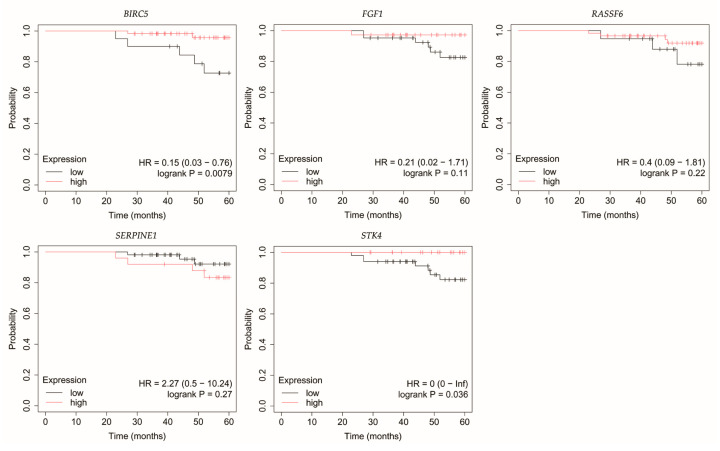
Overall survival of patients with HER2-positive luminal B breast cancer. *BIRC5*, baculoviral IAP repeat containing 5; *FGF1*, fibroblast growth factor 1; *RASSF6*, Ras association domain family member 6; *SERPINE1*, serpin family E member 1; *STK4*, serine/threonine kinase 4.

**Figure 6:**
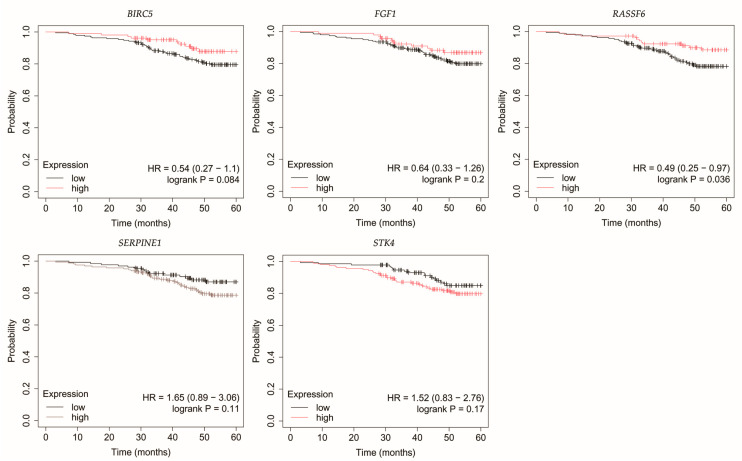
Overall survival of patients with non-luminal HER2-positive breast cancer. *BIRC5*, baculoviral IAP repeat containing 5; *FGF1*, fibroblast growth factor 1; *RASSF6*, Ras association domain family member 6; *SERPINE1*, serpin family E member 1; *STK4*, serine/threonine kinase 4.

**Figure 7:**
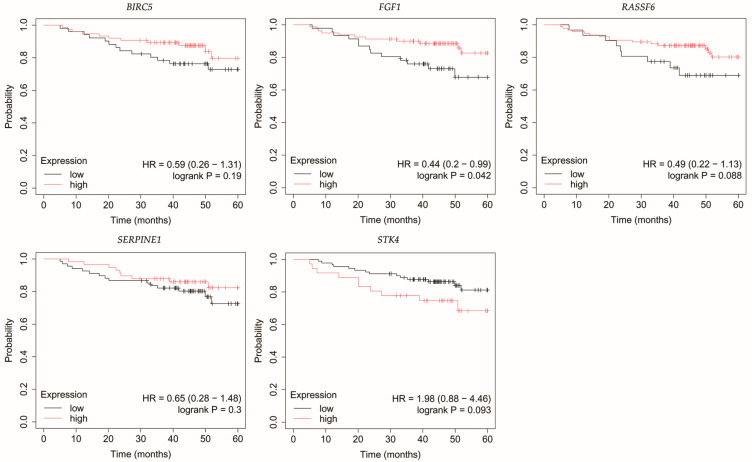
Overall survival analysis of patients with TNBC. *BIRC5*, baculoviral IAP repeat containing 5; *FGF1*, fibroblast growth factor 1; *FGF1*, fibroblast growth factor 1; *RASSF6*, Ras association domain family member 6; *SERPINE1*, serpin family E member 1; *STK4*, serine/threonine kinase 4.

The outcomes of the overall survival (OS) analysis are presented in Figure 3, Figure 4, Figure 5, Figure 6 and Figure 7.

In luminal A breast cancer, reduced expression of *RASSF6* and *STK4* correlated with poorer OS (Figure 3). Similarly, for *BIRC5*, however, it should be noted that *p* was 0.049.

Loss of *STK4* expression in HER2-negative luminal B tumors correlated with reduced OS (Figure 4).

In HER2-positive luminal B breast cancer, reduced *STK4* expression was correlated with decreased OS (Figure 5).

In non-luminal HER2-positive breast cancer, reduced *RASSF6* expression was linked with poorer OS (Figure 6).

Analysis of TNBC cases indicated that patients with low *FGF1* expression exhibited reduced OS (Figure 7).

## 4. Discussion

Through an integrated evaluation of five molecular subtypes, we identified a core set of Hippo-related genes that are consistently altered across breast cancers. We observed upregulation of *BIRC5*, and *SERPINE1*, along with *FGF1*, *RASSF6* and *STK4* downregulation, which was further confirmed at the protein level. Furthermore, we identified miRNAs potentially targeting *SERPINE1* and *STK4*.

STK4, also known as MST1, is a pivotal upstream kinase of the Hippo pathway responsible for phosphorylating and activating LATS1/2, thereby restricting YAP/TAZ nuclear translocation and oncogenic activity [22]. Its loss has been linked to tumor initiation and progression, suggesting a role as an early destabilizer of Hippo signaling [23]. Lin et al. confirmed the suppressive role of STK4 in breast cancer, emphasizing that its low levels were associated with reduced patient survival [24] and proposed its utility as a prognostic and predictive biomarker, as well as a therapeutic target [25]. Similarly, Jun et al. demonstrated that STK4 overexpression suppressed proliferation and migration of breast cancer cells, whereas the application of an STK4 inhibitor reversed these effects and inhibited the Hippo pathway [26]. Li et al. demonstrated that STK4 can undergo deacetylation, which leads to its degradation in lysosomes during autophagy. This resulted in decreased STK4 expression and promotion of breast cancer cell growth [27]. Our results align with these findings, as we observed consistent STK4 downregulation across all breast cancer subtypes. Bioinformatic predictions suggest that this effect may, at least in part, be mediated by miR-522-3p. Interestingly, this miRNA has been reported to exert both oncogenic and tumor-suppressive roles depending on the cancer context [28,29,30,31,32]. In breast cancer, bioinformatic analysis by Tan et al. revealed that miR-522 participates in the regulation of proliferation, migration, and EMT [33]. Dong et al. demonstrated that targeting p63 protein by this miRNA in breast epithelial cells resulted in the acquisition of mesenchymal morphology, which accelerated cell migration. Reversing this process may therefore constitute a potential anti-metastatic strategy [34]. In addition, Wang et al. showed that miR-522 can target BRCA1 in TNBC, where its overexpression predicted reduced overall patient survival and a higher incidence of lymph node metastasis [35]. Collectively, we have identified *STK4* as a potential target of miR-522-3p regardless of breast cancer subtype. Importantly, low *STK4* expression was linked to poorer overall survival in luminal A and luminal B subtypes, reinforcing its value as a prognostic biomarker and potential therapeutic target.

RASSF6 is recognized as a tumor suppressor that is downregulated in various cancers [36,37,38], including breast cancer [39]. Mechanistically, it interacts with MDM2, promoting its degradation and thereby stabilizing p53 [40]. Remarkably, even in the absence of p53, RASSF6 retains the ability to induce apoptosis and cell cycle arrest through the pRb-E2F1 axis [41]. Moreover, RASSF6 directly activates the Hippo pathway by enhancing the phosphorylation of STK4/3 and LATS1/2 kinases [39], thereby reinforcing its role as a critical upstream activator of Hippo tumor-suppressive signaling. In our study, RASSF6 was consistently downregulated across all breast cancer subtypes, indicating a subtype-independent mechanism for Hippo pathway impairment. Unlike *STK4*, this effect was not attributable to any of the miRNAs identified in our analysis. Clinically, low *RASSF6* expression correlated with poorer overall survival in patients with luminal A and non-luminal HER2-positive cancers, underscoring its prognostic significance and potential utility as a biomarker of Hippo pathway integrity.

FGF1 can influence the Hippo pathway through interactions with receptor tyrosine kinases, notably FGFR1, which subsequently activate downstream MAPK and PI3K signaling cascades [42,43,44]. This signaling cross-talk represents a complex regulatory network in which FGF1 binding induces downstream changes that impact cell proliferation, survival, and differentiation, which are also modulated by the Hippo pathway [42,45]. The FGF1/FGFR axis has been linked to breast cell proliferation and migration, suggesting its potential as a therapeutic target [46]. Szymczak et al. confirmed the involvement of FGF1 in drug resistance in the MCF-7 breast cancer line [47]. Castillo-Castrejon et al. demonstrated that FGF1 can activate ER independently of estrogen in obese women, promoting breast cancer progression [48]. In our study, FGF1 expression was decreased across all subtypes, with low levels correlating with worse overall survival in TNBC. Notably, no miRNAs meeting our selection criteria were predicted to target *FGF1*, indicating that alternative regulatory mechanisms may be responsible. Overall, FGF1 downregulation may disrupt upstream extracellular signaling that modulates Hippo activity, further contributing to pathway suppression.

BIRC5, also known as survivin, belongs to the family of antiapoptotic proteins whose expression can be induced by YAP/TAZ, resulting in inhibition of apoptosis [49]. It promotes tumorigenesis through two principal mechanisms: preventing apoptosis via caspase inhibition, which allows cancer cells to survive, and facilitating mitotic progression, thereby supporting cancer cell survival and proliferation [50]. Overexpression of BIRC5 is frequently observed in various cancers and is linked to tumor initiation and progression [51], making it a valuable diagnostic and prognostic biomarker as well as a potential therapeutic target [52]. In breast cancer, high BIRC5 expression has been reported particularly in basal-like and luminal B subtypes, correlating with poorer patient survival independent of estrogen receptor status or lymph node involvement [53]. Targeting BIRC5 in combination with standard therapies has been suggested by Mehraj et al. as a strategy to improve treatment efficacy [54]. Hamilton et al. emphasized higher risk of breast cancer tumors with BIRC5 overexpression in Black and younger women [55]. Al-Yahya et al. demonstrated that post-transcriptional regulation by RNA-binding proteins, such as tristetraprolin, can reduce BIRC5 levels, inhibiting proliferation and enhancing patient outcomes [56]. Consistent with previous findings, our analysis revealed BIRC5 overexpression across all breast cancer subtypes, reflecting a functional consequence of Hippo pathway inactivation that promotes tumor cell survival. Importantly, *BIRC5* expression in our cohort was not significantly influenced by miRNAs, suggesting that transcriptional activation (likely via YAP/TAZ–TEAD complexes) is the predominant mechanism. This consistent upregulation positions BIRC5 as a central Hippo effector and a promising therapeutic target, especially in contexts of apoptosis-resistant tumors.

SERPINE1, also known as PAI-1, is a serine protease inhibitor involved in extracellular matrix remodeling, angiogenesis, and metastatic progression [57]. It is a recognized transcriptional target of YAP/TAZ [58] and is frequently upregulated across multiple cancer types, where its overexpression correlates with tumor promotion and poor patient prognosis [57,59,60,61]. In breast cancer specifically, elevated SERPINE1 levels have been associated with worse overall and relapse-free survival [62]. Zhang et al. demonstrated a link between high SERPINE1 levels and paclitaxel resistance in TNBC [63], whereas Su et al. reported similar observation for obesity-associated tumor radioresistance [64]. In our study, SERPINE1 was consistently overexpressed across all breast cancer subtypes, underscoring its role as both a Hippo pathway effector and a contributor to tumor progression. Notably, increased *SERPINE1* expression correlated with decreased levels of miR-199b-5p and miR-30a-3p, both of which are established tumor suppressor miRNAs in breast cancer [65,66,67,68,69,70,71,72,73]. This suggests that SERPINE1 dysregulation may be reinforced at multiple regulatory levels, and our findings indicate it as a novel target of miR-199b-5p and miR-30a-3p, highlighting potential avenues for therapeutic intervention.

Although the identified genes did not display pronounced heterogeneity in expression or prognosis between different breast cancer subtypes, this lack of variation highlights their potential as ‘subtype-independent’ biomarkers. Rather than distinguishing between subtypes, these genes may reflect a core dysregulation of the Hippo pathway across breast cancers. Such markers may complement existing subtype-specific classifiers by offering universal prognostic and therapeutic insights, applicable irrespective of molecular subtype.

Our study has several limitations. First, the cohort consisted exclusively of Polish women with T1N0M0 tumors, which may restrict generalizability to other populations. Second, the numbers of patients in the non-luminal HER2-positive and TNBC subgroups were relatively small compared with luminal subtypes, which may affect the robustness of subgroup-specific findings. Third, protein expression was validated using ELISA rather than Western blot; while ELISA provides quantitative data, it does not allow visualization of full-length protein bands. Fourth, functional validation of the predicted miRNA–mRNA interactions (e.g., luciferase reporter assays, knockdown/overexpression experiments) was not performed, and future studies are required to confirm these regulatory relationships.

## 5. Conclusions

This study reveals a conserved pattern of Hippo pathway dysregulation across all major breast cancer subtypes. We demonstrated that STK4, RASSF6, and FGF1 were consistently downregulated, whereas BIRC5, and SERPINE1 were upregulated. These changes reflect both impaired upstream Hippo activation and enhanced downstream oncogenic signaling. Notably, *STK4* and *SERPINE1* appear to be regulated by miRNAs, including miR-522-3p, miR-199b-5p, and miR-30a-3p. By focusing on subtype-independent events, our findings highlight the Hippo pathway as a common axis of dysregulation in breast cancer. This suggests that Hippo-related genes and their miRNA regulators could serve as universal biomarkers or therapeutic targets, complementing subtype-specific treatment approaches.

## Figures and Tables

**Table 1:** Patients information.

Cancer Subtype	Cancer Grade			Age		BMI [kg/m^2^]
	G1	G2	G3	<50 years	>50 years	
Luminal A	23	48	59	43	87	30.8 ± 2.8
HER2-negative luminal B	31	57	12	32	68	30.2 ± 4.6
HER2-positive luminal B	23	57	16	19	77	32.1 ± 6.2
Non-luminal HER2-positive	9	12	15	9	27	33.2 ± 5.7
TNBC	14	21	8	10	33	34.7 ± 3

HER2, human epidermal growth factor receptor 2; TNBC, triple-negative breast cancer; BMI, body mass index.

**Table 2:** RT-qPCR primer details.

mRNA	RT-qPCR Primers (5′–3′)
*BIRC5*	Forward: CCACTGAGAACGAGCCAGACTT Reverse: GTATTACAGGCGTAAGCCACCG
*FGF1*	Forward: ATGGCACAGTGGATGGGACAAG Reverse: TAAAAGCCCGTCGGTGTCCATG
*RASSF6*	Forward: CGTATTAGTGAGCTGGACAGGAC Reverse: CTGGTTCATCCTTTGCATGTGGC
*SERPINE1*	Forward: CTCATCAGCCACTGGAAAGGCA Reverse: GACTCGTGAAGTCAGCCTGAAAC
*STK4*	Forward: CTGTGTAGCAGACATCTGGTCC Reverse: CTGGTTTTCGGAATGTGGGAGG
*ACTB*	Forward: TCACCCACACTGTGCCCATCTACGA Reverse: CAGCGGAACCGCTCATTGCCAATGG

*BIRC5*, baculoviral IAP repeat containing 5; *FGF1*, fibroblast growth factor 1; *RASSF6*, Ras association domain family member 6; *SERPINE1*, serpin family E member 1; *STK4*, serine/threonine kinase 4; *ACTB*, β-actin.

**Table 3:** mRNAs from the Hippo signaling pathway that distinguish breast cancer from the control, regardless of the subtype (*p* < 0.05; FC > 2 or <−2).

ID	mRNA	Fold Change				
		LumA vs. C	HER2- Negative LumB vs. C	HER2- Positive LumB vs. C	Non-Luminal HER2-Positive vs. C	TNBC vs. C
202095_s_at	*BIRC5*	2.11	2.99	3.49	3.77	4.33
205117_at	*FGF1*	−2.46	−3.21	−3.2	−3.91	−4.52
233463_at	*RASSF6*	−2.47	−2.97	−3.13	−3.27	−3.54
235638_at		−3.02	−3.33	−3.51	−3.4	−3.95
202627_s_at	*SERPINE1*	3.17	2.68	2.77	3.36	3.97
202628_s_at		3.21	2.43	2.87	3.5	3.52
1568765_at		2.88	3.1	3.07	3.49	3.57
211085_s_at	*STK4*	−2.02	−2.22	−2.25	−2.63	−3.75

ID, number of the probe; LumA, luminal A; LumB, luminal B; HER2, human epidermal growth factor receptor 2; TNBC, triple-negative breast cancer; C, control; *BIRC5*, baculoviral IAP repeat containing 5; *FGF1*, fibroblast growth factor 1; *RASSF6*, Ras association domain family member 6; *SERPINE1*, serpin family E member 1; *STK4*, serine/threonine kinase 4.

**Table 4:** Concentrations of selected proteins in breast cancer subtypes and the control group (*p* < 0.05).

Protein [ng/mL]	Control	LumA	HER2-Negative LumB	HER2-Positive LumB	HER2-Positive	TNBC
BIRC5	1.13 ± 0.2	2.94 ± 0.2 *	3.93 ± 0.3 *	3.98 ± 0.3 *	4.08 ± 0.3 *	6.47 ± 0.3 *
FGF1	1.27 ± 0.1	below detection threshold *	below detection threshold *	below detection threshold *	below detection threshold *	below detection threshold *
RASSF6	0.32 ± 0.1	below detection threshold *	below detection threshold *	below detection threshold *	below detection threshold *	below detection threshold *
SERPINE1	6.23 ± 0.2	7.3 ± 0.1 *	8.43 ± 0.2 *	8.79 ± 0.2 *	11.48 ± 0.2 *	13.05 ± 0.2 *
STK4	2.34 ± 0.4	below detection threshold *	below detection threshold *	below detection threshold *	below detection threshold *	below detection threshold *

LumA, luminal A; LumB, luminal B; HER2, human epidermal growth factor receptor 2; TNBC, triple-negative breast cancer; BIRC5, baculoviral IAP repeat containing 5; FGF1, fibroblast growth factor 1; RASSF6, Ras association domain family member 6; SERPINE1, serpin family E member 1; STK4, serine/threonine kinase 4. * *p* < 0.05 vs. control.

**Table 5:** miRNAs with potential regulatory roles on the investigated genes (*p* < 0.05; FC > 2 or <−2).

mRNA	miRNA	Target Score	Fold Change				
			LumA vs. C	HER2-Negative LumB vs. C	HER2-Positive LumB vs. C	HER2-Positive vs. C	TNBC vs. C
*SERPINE1*	miR-199b-5p	86	−2.17	−2.36	−3.03	−3.64	−3.41
	miR-30a-3p	80	−2.69	−3.04	−4.19	−4.44	−5.28
*STK4*	miR-522-3p	89	2.05	2.17	2.38	2.33	2.56

LumA, luminal A; LumB, luminal B; HER2, human epidermal growth factor receptor 2; TNBC, triple-negative breast cancer; C, control; *SERPINE1*, serpin family E member 1; *STK4*, serine/threonine kinase 4.

## Data Availability

The data used to support findings of this study are included in this article. The data will not be shared due to third-party rights and commercial confidentiality.

## Abbreviations

The following abbreviations are used in this manuscript:

ACTB	β-actin
BIRC5	baculoviral IAP repeat containing 5
ELISA	Enzyme-Linked Immunosorbent Assay
EMT	epithelial–mesenchymal transition
FGF1	fibroblast growth factor 1
LATS1	large tumor suppressor kinase 1
LATS2	large tumor suppressor kinase 2
OS	Overall survival
RASSF6	Ras association domain family member 6
RT-qPCR	Reverse Transcription Quantitative Polymerase Chain Reaction
SAV1	Salvador
SERPINE1	serpin family E member 1
STK3	serine/threonine-protein kinase 3
STK4	serine/threonine-protein kinase 4
TAZ	transcriptional coactivator with PDZ-binding motif
TEAD	transcriptional enhanced associate domain
TNBC	triple-negative breast cancer
YAP	Yes-associated protein

## References

[1] Bray F., Laversanne M., Sung H., Ferlay J., Siegel R.L., Soerjomataram I., Jemal A. (2024). Global Cancer Statistics 2022: GLOBOCAN Estimates of Incidence and Mortality Worldwide for 36 Cancers in 185 Countries. CA Cancer J. Clin..

[2] Gąska I., Czerw A., Pajewska M., Partyka O., Deptała A., Badowska-Kozakiewicz A., Budzik M., Sygit K., Wojtyła-Buciora P., Drobnik J. (2025). The Cost of Breast Cancer: Economic and Social Perspective. Cancers.

[3] Roy M., Fowler A.M., Ulaner G.A., Mahajan A. (2023). Molecular Classification of Breast Cancer. PET Clin..

[4] Fu M., Hu Y., Lan T., Guan K.-L., Luo T., Luo M. (2022). The Hippo Signalling Pathway and Its Implications in Human Health and Diseases. Signal Transduct. Target. Ther..

[5] Ma S., Meng Z., Chen R., Guan K.-L. (2019). The Hippo Pathway: Biology and Pathophysiology. Annu. Rev. Biochem..

[6] Sebio A., Lenz H.-J. (2015). Molecular Pathways: Hippo Signaling, a Critical Tumor Suppressor. Clin. Cancer Res..

[7] Ghaboura N. (2025). Unraveling the Hippo Pathway: YAP/TAZ as Central Players in Cancer Metastasis and Drug Resistance. EXCLI J..

[8] Misra J.R., Irvine K.D. (2018). The Hippo Signaling Network and Its Biological Functions. Annu. Rev. Genet..

[9] Zheng Y., Pan D. (2019). The Hippo Signaling Pathway in Development and Disease. Dev. Cell.

[10] Zhong Z., Jiao Z., Yu F.-X. (2024). The Hippo Signaling Pathway in Development and Regeneration. Cell Rep..

[11] Di X., Gao X., Peng L., Ai J., Jin X., Qi S., Li H., Wang K., Luo D. (2023). Cellular Mechanotransduction in Health and Diseases: From Molecular Mechanism to Therapeutic Targets. Signal Transduct. Target. Ther..

[12] Jiang L., Li J., Zhang C., Shang Y., Lin J. (2020). YAP-mediated Crosstalk Between the Wnt and Hippo Signaling Pathways (Review). Mol. Med. Rep..

[13] Zhang C., Wei W., Tu S., Liang B., Li C., Li Y., Luo W., Wu Y., Dai X., Wang Y. (2024). Upregulation of CYR61 by TGF-β and YAP Signaling Exerts a Counter-Suppression of Hepatocellular Carcinoma. J. Biol. Chem..

[14] Borreguero-Muñoz N., Fletcher G.C., Aguilar-Aragon M., Elbediwy A., Vincent-Mistiaen Z.I., Thompson B.J. (2019). The Hippo Pathway Integrates PI3K–Akt Signals with Mechanical and Polarity Cues to Control Tissue Growth. PLoS Biol..

[15] Paul S., Hagenbeek T.J., Tremblay J., Kameswaran V., Ong C., Liu C., Guarnaccia A.D., Mondo J.A., Hsu P.L., Kljavin N.M. (2025). Cooperation Between the Hippo and MAPK Pathway Activation Drives Acquired Resistance to TEAD Inhibition. Nat. Commun..

[16] Kandettu A., Radhakrishnan R., Chakrabarty S., Sriharikrishnaa S., Kabekkodu S.P. (2020). The Emerging Role of miRNA Clusters in Breast Cancer Progression. Biochim. Et Biophys. Acta (BBA)—Rev. Cancer.

[17] Sirek T., Sirek A., Zmarzły N., Opławski M., Król-Jatręga K., Boroń D., Chalcarz M., Ossowski P., Dziobek K., Strojny D. (2025). Impact of MiRNAs on Wnt-Related Gene Activity in Breast Cancer. Sci. Rep..

[18] Chen Y., Wang X. (2020). miRDB: An Online Database for Prediction of Functional microRNA Targets. Nucleic Acids Res..

[19] Győrffy B. (2024). Integrated Analysis of Public Datasets for the Discovery and Validation of Survival-Associated Genes in Solid Tumors. Innovation.

[20] Győrffy B. (2024). Transcriptome-Level Discovery of Survival-Associated Biomarkers and Therapy Targets in Non-Small-Cell Lung Cancer. Br. J. Pharmacol..

[21] Faul F., Erdfelder E., Lang A.-G., Buchner A. (2007). G*Power 3: A Flexible Statistical Power Analysis Program for the Social, Behavioral, and Biomedical Sciences. Behav. Res. Methods.

[22] Meng Z., Moroishi T., Guan K.-L. (2016). Mechanisms of Hippo Pathway Regulation. Genes. Dev..

[23] Zhou D., Conrad C., Xia F., Park J.-S., Payer B., Yin Y., Lauwers G.Y., Thasler W., Lee J.T., Avruch J. (2009). Mst1 and Mst2 Maintain Hepatocyte Quiescence and Suppress the Development of Hepatocellular Carcinoma Through Inactivation of the Yap1 Oncogene. Cancer Cell.

[24] Lin X., Cai F., Li X., Kong X., Xu C., Zuo X., Yang Q. (2013). Prognostic Significance of Mammalian Sterile 20-like Kinase 1 in Breast Cancer. Tumour Biol..

[25] Lin X.-Y., Cai F.-F., Wang M.-H., Pan X., Wang F., Cai L., Cui R.-R., Chen S., Biskup E. (2017). Mammalian Sterile 20-like Kinase 1 Expression and Its Prognostic Significance in Patients with Breast Cancer. Oncol. Lett..

[26] Jin X., Zhu L., Xiao S., Cui Z., Tang J., Yu J., Xie M. (2021). MST1 Inhibits the Progression of Breast Cancer by Regulating the Hippo Signaling Pathway and May Serve as a Prognostic Biomarker. Mol. Med. Rep..

[27] Li L., Fang R., Liu B., Shi H., Wang Y., Zhang W., Zhang X., Ye L. (2016). Deacetylation of Tumor-Suppressor MST1 in Hippo Pathway Induces Its Degradation Through HBXIP-Elevated HDAC6 in Promotion of Breast Cancer Growth. Oncogene.

[28] Ma G., Xue W., Ni J., Tao R. (2022). MiR-522-3p Targets Transcription Factor 4 to Overcome Cisplatin Resistance of Gastric Cells. J. Oncol..

[29] Miyamoto M., Sawada K., Nakamura K., Yoshimura A., Ishida K., Kobayashi M., Shimizu A., Yamamoto M., Kodama M., Hashimoto K. (2020). Paclitaxel Exposure Downregulates miR-522 Expression and Its Downregulation Induces Paclitaxel Resistance in Ovarian Cancer Cells. Sci. Rep..

[30] Zhang L., Zhang P., Tan Y., Feng Q., Zhao R. (2021). MicroRNA-522-3p Plays an Oncogenic Role in Glioblastoma Through Activating Wnt/β-Catenin Signaling Pathway via Targeting SFRP2. Neuroreport.

[31] Liu Q., Bao H., Zhang S., Li C., Sun G., Sun X., Fu T., Wang Y., Liang P. (2024). MicroRNA-522-3p Promotes Brain Metastasis in Non-Small Cell Lung Cancer by Targeting Tensin 1 and Modulating Blood-Brain Barrier Permeability. Exp. Cell Res..

[32] Zhang J., Pan Y., Jin L., Yang H., Cao P. (2024). Exosomal-miR-522-3p Derived from Cancer-Associated Fibroblasts Accelerates Tumor Metastasis and Angiogenesis via Repression Bone Morphogenetic Protein 5 in Colorectal Cancer. J. Gastroenterol. Hepatol..

[33] Tan S.M., Kirchner R., Jin J., Hofmann O., McReynolds L., Hide W., Lieberman J. (2014). Sequencing of Captive Target Transcripts Identifies the Network of Regulated Genes and Functions of Primate-Specific miR-522. Cell Rep..

[34] Dong Y., Long J., Luo X., Xie G., Xiao Z.J., Tong Y. (2021). Targeting of ΔNp63α by miR-522 Promotes the Migration of Breast Epithelial Cells. FEBS Open Bio.

[35] Wang W., Zhang W., Wu J., Zhou Z., Ma J. (2022). miR-522 Regulates Cell Proliferation, Migration, Invasion Capacities and Acts as a Potential Biomarker to Predict Prognosis in Triple-Negative Breast Cancer. Clin. Exp. Med..

[36] Wen Y., Wang Q., Zhou C., Yan D., Qiu G., Yang C., Tang H., Peng Z. (2011). Decreased Expression of RASSF6 Is a Novel Independent Prognostic Marker of a Worse Outcome in Gastric Cancer Patients after Curative Surgery. Ann. Surg. Oncol..

[37] Ye H.-L., Li D.-D., Lin Q., Zhou Y., Zhou Q.-B., Zeng B., Fu Z.-Q., Gao W.-C., Liu Y.-M., Chen R.-W. (2015). Low RASSF6 Expression in Pancreatic Ductal Adenocarcinoma Is Associated with Poor Survival. World J. Gastroenterol..

[38] Tan S., Bian X., Wu B., Chen X. (2019). RASSF6 Is Downregulated In Human Bladder Cancers And Regulates Doxorubicin Sensitivity And Mitochondrial Membrane Potential via the Hippo Signaling Pathway. Onco Targets Ther..

[39] He Z., Zhao T.-T., Jin F., Li J.-G., Xu Y.-Y., Dong H.-T., Liu Q., Xing P., Zhu G.-L., Xu H. (2018). Downregulation of RASSF6 Promotes Breast Cancer Growth and Chemoresistance Through Regulation of Hippo Signaling. Biochem. Biophys. Res. Commun..

[40] Sarkar A., Iwasa H., Hossain S., Xu X., Sawada T., Shimizu T., Maruyama J., Arimoto-Matsuzaki K., Hata Y. (2017). Domain Analysis of Ras-Association Domain Family Member 6 upon Interaction with MDM2. FEBS Lett..

[41] Hossain S., Iwasa H., Sarkar A., Maruyama J., Arimoto-Matsuzaki K., Hata Y. (2018). The RASSF6 Tumor Suppressor Protein Regulates Apoptosis and Cell Cycle Progression via Retinoblastoma Protein. Mol. Cell Biol..

[42] Teven C.M., Farina E.M., Rivas J., Reid R.R. (2014). Fibroblast Growth Factor (FGF) Signaling in Development and Skeletal Diseases. Genes. Dis..

[43] Zakrzewska M., Opalinski L., Haugsten E.M., Otlewski J., Wiedlocha A. (2019). Crosstalk Between P38 and Erk 1/2 in Downregulation of FGF1-Induced Signaling. Int. J. Mol. Sci..

[44] Jeong S.-H., Kim H.-B., Kim M.-C., Lee J., Lee J.H., Kim J.-H., Kim J.-W., Park W.-Y., Kim S.-Y., Kim J.B. (2018). Hippo-Mediated Suppression of IRS2/AKT Signaling Prevents Hepatic Steatosis and Liver Cancer. J. Clin. Investig..

[45] Ferguson H.R., Smith M.P., Francavilla C. (2021). Fibroblast Growth Factor Receptors (FGFRs) and Noncanonical Partners in Cancer Signaling. Cells.

[46] Gao Y., Wang Y., Yu J., Guo R. (2022). FGF Exhibits an Important Biological Role on Regulating Cell Proliferation of Breast Cancer When It Transports Into The Cell Nuclei. Cell Biochem. Biophys..

[47] Szymczyk J., Czyrek A., Otlewski J., Zakrzewska M. (2023). FGF1 Protects MCF-7 Cells against Taltobulin Through Both the MEKs/ERKs and PI3K/AKT Signaling Pathway. Biomedicines.

[48] Castillo-Castrejon M., Sankofi B.M., Murguia S.J., Udeme A.-A., Cen H.H., Xia Y.H., Thomas N.S., Berry W.L., Jones K.L., Richard V.R. (2023). FGF1 Supports Glycolytic Metabolism Through the Estrogen Receptor in Endocrine-Resistant and Obesity-Associated Breast Cancer. Breast Cancer Res..

[49] Lee H., Cho S.W., Cha H.S., Tae K., Choi C.Y. (2025). Transient Activation of YAP/TAZ Confers Resistance to Morusin-Induced Apoptosis. BMC Mol. Cell Biol..

[50] Wang Q., Greene M.I. (2024). Survivin as a Therapeutic Target for the Treatment of Human Cancer. Cancers.

[51] Fäldt Beding A., Larsson P., Helou K., Einbeigi Z., Parris T.Z. (2022). Pan-Cancer Analysis Identifies BIRC5 as a Prognostic Biomarker. BMC Cancer.

[52] Li G., Wang Y., Wang W., Lv G., Li X., Wang J., Liu X., Yuan D., Deng S., You D. (2024). BIRC5 as a Prognostic and Diagnostic Biomarker in Pan-Cancer: An Integrated Analysis of Expression, Immune Subtypes, and Functional Networks. Front. Genet..

[53] Oparina N., Erlandsson M.C., Fäldt Beding A., Parris T., Helou K., Karlsson P., Einbeigi Z., Bokarewa M.I. (2021). Prognostic Significance of BIRC5/Survivin in Breast Cancer: Results from Three Independent Cohorts. Cancers.

[54] Mehraj U., Aisha S., Sofi S., Mir M.A. (2022). Expression Pattern and Prognostic Significance of Baculoviral Inhibitor of Apoptosis Repeat-Containing 5 (BIRC5) in Breast Cancer: A Comprehensive Analysis. Adv. Cancer Biol.—Metastasis.

[55] Hamilton A.M., Walens A., Van Alsten S.C., Olsson L.T., Nsonwu-Farley J., Gao X., Kirk E.L., Perou C.M., Carey L.A., Troester M.A. (2024). BIRC5 Expression by Race, Age and Clinical Factors in Breast Cancer Patients. Breast Cancer Res..

[56] Al-Yahya S., Al-Saif M., Al-Ghamdi M., Moghrabi W., Khabar K.S.A., Al-Souhibani N. (2023). Post-Transcriptional Regulation of BIRC5/Survivin Expression and Induction of Apoptosis in Breast Cancer Cells by Tristetraprolin. RNA Biol..

[57] Mathews S.G., Krishna R.B.D., M L., K N., Murali S., Agarwal P., Rani E., F A.M. (2024). The Role of the Plasminogen Activator Inhibitor 1 (PAI1) in Ovarian Cancer: Mechanisms and Therapeutic Implications. Glob. Med. Genet..

[58] Kong H.-J., Kwon E.-J., Kwon O.-S., Lee H., Choi J.-Y., Kim Y.-J., Kim W., Cha H.-J. (2021). Crosstalk Between YAP and TGFβ Regulates SERPINE1 Expression in Mesenchymal Lung Cancer Cells. Int. J. Oncol..

[59] Chen S., Li Y., Zhu Y., Fei J., Song L., Sun G., Guo L., Li X. (2022). SERPINE1 Overexpression Promotes Malignant Progression and Poor Prognosis of Gastric Cancer. J. Oncol..

[60] Polo-Generelo S., Rodríguez-Mateo C., Torres B., Pintor-Tortolero J., Guerrero-Martínez J.A., König J., Vázquez J., Bonzón-Kulichenco E., Padillo-Ruiz J., de la Portilla F. (2024). Serpine1 mRNA Confers Mesenchymal Characteristics to the Cell and Promotes CD8+ T Cells Exclusion from Colon Adenocarcinomas. Cell Death Discov..

[61] Liu Y., Li X., Chen S., Zhu C., Shi Y., Dang S., Zhang W., Li W. (2023). Pan-Cancer Analysis of SERPINE Family Genes as Biomarkers of Cancer Prognosis and Response to Therapy. Front. Mol. Biosci..

[62] Ferroni P., Roselli M., Portarena I., Formica V., Riondino S., LA Farina F., Costarelli L., Melino A., Massimiani G., Cavaliere F. (2014). Plasma Plasminogen Activator Inhibitor-1 (PAI-1) Levels in Breast Cancer—Relationship with Clinical Outcome. Anticancer. Res..

[63] Zhang Q., Lei L., Jing D. (2020). Knockdown of SERPINE1 Reverses Resistance of Triple-negative Breast Cancer to Paclitaxel via Suppression of VEGFA. Oncol. Rep..

[64] Su Y.-H., Wu Y.-Z., Ann D.K., Chen J.L.-Y., Kuo C.-Y. (2023). Obesity Promotes Radioresistance Through SERPINE1-Mediated Aggressiveness and DNA Repair of Triple-Negative Breast Cancer. Cell Death Dis..

[65] Fang C., Zhao Y., Guo B. (2013). MiR-199b-5p Targets HER2 in Breast Cancer Cells. J. Cell Biochem..

[66] Fang C., Wang F.-B., Li Y., Zeng X.-T. (2016). Down-Regulation of miR-199b-5p Is Correlated with Poor Prognosis for Breast Cancer Patients. Biomed. Pharmacother..

[67] Wu A., Chen Y., Liu Y., Lai Y., Liu D. (2018). miR-199b-5p Inhibits Triple Negative Breast Cancer Cell Proliferation, Migration and Invasion by Targeting DDR1. Oncol. Lett..

[68] Lin X., Qiu W., Xiao Y., Ma J., Xu F., Zhang K., Gao Y., Chen Q., Li Y., Li H. (2019). MiR-199b-5p Suppresses Tumor Angiogenesis Mediated by Vascular Endothelial Cells in Breast Cancer by Targeting ALK1. Front. Genet..

[69] Ruan X., Yan W., Cao M., Daza R.A.M., Fong M.Y., Yang K., Wu J., Liu X., Palomares M., Wu X. (2024). Breast Cancer Cell-Secreted miR-199b-5p Hijacks Neurometabolic Coupling to Promote Brain Metastasis. Nat. Commun..

[70] Chang C.-W., Yu J.-C., Hsieh Y.-H., Yao C.-C., Chao J.-I., Chen P.-M., Hsieh H.-Y., Hsiung C.-N., Chu H.-W., Shen C.-Y. (2016). MicroRNA-30a Increases Tight Junction Protein Expression to Suppress the Epithelial-Mesenchymal Transition and Metastasis by Targeting Slug in Breast Cancer. Oncotarget.

[71] Zhang H.-D., Jiang L.-H., Sun D.-W., Li J., Tang J.-H. (2017). miR-30a Inhibits the Biological Function of Breast Cancer Cells by Targeting Notch1. Int. J. Mol. Med..

[72] Xiao B., Shi X., Bai J. (2019). miR-30a Regulates the Proliferation and Invasion of Breast Cancer Cells by Targeting Snail. Oncol. Lett..

[73] Mitsueda R., Nagata A., Toda H., Tomioka Y., Yasudome R., Kato M., Shinden Y., Nakajo A., Seki N. (2024). Identification of Tumor-Suppressive miR-30a-3p Controlled Genes: ANLN as a Therapeutic Target in Breast Cancer. Noncoding RNA.

